# Stage-variations of anandamide hydrolase activity in the mouse uterus during the natural oestrus cycle

**DOI:** 10.1186/1743-1050-3-3

**Published:** 2006-03-30

**Authors:** Francesca G Klinger, Natalia Battista, Massimo De Felici, Mauro Maccarrone

**Affiliations:** 1 Department of Public Health and Cell Biology, University of Rome Tor Vergata, 00133 Rome, Italy; 2 Department of Biomedical Sciences, University of Teramo, 64100 Teramo, Italy; 3 IRCCS C. Mondino, Mondino-Tor Vergata Center for Experimental Neurobiology, 00133 Rome, Italy

## Abstract

Recent studies have demonstrated that the endogenous cannabinoids are important modulators of fertility in mammals. In particular, a role of the endocannabinoid system in early stages of embryo development, oviductal transport of embryos, pregnancy maintenance and labour has been demonstrated in rodents and/or in humans. In the present paper, we report the analysis of FAAH activity and protein content in the mouse uterus as a function of the natural oestrus cycle stages. Variations of FAAH activity are discussed in relationship to changes in sex steroid levels and to the possible action of AEA on remodelling of uterine tissues.

## Introduction

Recent studies have demonstrated that the endogenous cannabinoids (bioactive fatty acid amides and esters termed endocannabinoids) are important modulators of fertility in mammals [1,2]. The endocannabinoid anandamide (*N*-arachidonoylethanolamine, AEA) and congeners, cannabinoid CB1 and CB2 receptors, the AEA-synthesizing enzymes *N*-acetyltransferase (NAT) and *N*-acylphosphatidylethanolamine (NAPE)-specific phospholipase D, the AEA membrane transporter (AMT) and the AEA-hydrolyzing enzyme fatty acid amide hydrolase (FAAH), form the endocannabinoid system. A role of this system in early stages of embryo development [2], oviductal transport of embryos [3], pregnancy maintenance and labour [4,5], has been demonstrated in rodents and/or in humans.

During early pregnancy, a proper endocannabinoid signalling in the uterus and in the preimplantation embryo (both expressing CB1 receptor, AMT and FAAH), and possibly also in the circulating immune cells, appears to be crucial for embryo development and implantation [2]. In particular, high levels of AEA cause inhibition of trophoblast proliferation at the interimplantation sites and induce blastocyst apoptosis, while low levels of AEA at the implantation sites favour trophoblast differentiation and outgrowth [4,6-8]. In rodents, variation of AEA levels in the pregnant uterus has been directly correlated with FAAH expression and activity in uterine tissues [4,9,10]. Interestingly, FAAH activity and expression of at the mRNA level can be modulated in the pregnant uterus by the blastocyst itself, and by sex hormones [4,10,11]. Besides pregnant uterus and preimplantation embryo, oviduct is also a target for endocannabinoid action. In fact, in the mouse CB1 deficiency causes early pregnancy loss due to retention of embryos in the oviduct [3].

In the present paper, we report the analysis of FAAH activity and protein content in the mouse uterus as a function of the natural oestrous cycle stages. Variations of FAAH activity are discussed in relationship to changes in sex steroid levels and to the possible action of AEA on remodelling of uterine tissues.

## Methods

### Uterine tissue collection during the oestrous cycle

The 4–5 day oestrous cycle of mouse was monitored by the examination of vaginal smears and classified in five stages; proestrus, oestrus, early or late-metoestrus and dioestrus [12]. CD-1 mice were killed by cervical dislocation and uterus quickly removed in M2 without BSA [4], cut in small pieces and rapidly frozen and stored at -70°C.

### Assay of FAAH activity and protein content

The hydrolysis of 10 μM [^3^H] AEA (223 Ci/mmol, from Perkin Elmer Life Sciences, Boston, MA) by the fatty acid amide hydrolase (E.C. 3.5.1.4; FAAH) activity was assayed in uterine extracts (20 μg/test)[4], by measuring the release of [^3^H] arachidonic acid, at pH 9.0, by reversed phase high performance liquid chromatography [4]. FAAH activity was expressed as pmol arachidonate released per min per mg protein.

FAAH protein content was determined in uterine homogenates (20 μg/test) by enzyme-linked immunosorbent assay (ELISA), performed as reported [13]. For the ELISA test rabbit anti-FAAH polyclonal antibodies [13] were prepared by Primm S.r.l. (Milan, Italy) and were used as first antibody (diluted 1:300), whereas goat anti-rabbit alkaline phosphatase conjugates (Bio-Rad Laboratories, Hercules, CA) were used as second antibodies (diluted 1:2000). Color development of the alkaline phsphatase reaction was measured at 405 nm, using *p*-nitrophenyl phosphate as substrate, and the ELISA test was linear in the range 0–50 μg/well of homogenate [13].

Data reported in this paper are the means ± S.D. of at least four independent experiments, each performed in duplicate. Statistical analysis was performed by the nonparametric Mann-Whitney U test, elaborating experimental data by means of the InStat 4 program (GraphPAD Software for Science, San Diego, CA).

## Findings

The uterus undergoes cellular remodelling during each sexual cycle, in order to be ready for a possible pregnancy. In the absence of pregnancy, uterine changes are reversible permitting preparation in a subsequent cycle. In the event of mating and successful fertilization, however, the changes in the uterus take another route to support pregnancy. The uterine cellular changes during cycle and pregnancy are regulated by the circulating levels of ovarian sex steroids estradiol (E2) and progesterone (P).

**Figure 1:**
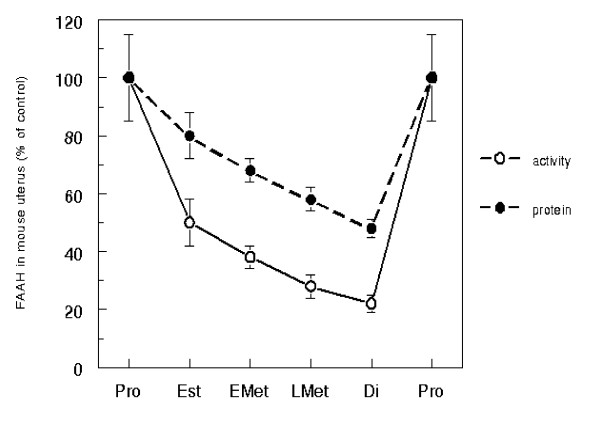
*Fluctuations of uterine FAAH in the normal oestrous cycle of mouse*. FAAH activity and protein content were measured as reported in "Materials and Methods", and were expressed as percentage of the proestrus value (100% = 250 ± 30 pmol/min per mg protein, for the activity; 0.370 ± 0.030 absorbance units at 405 nm, for the protein content). Vertical bars represent S.D. values. Pro, proestrus; Est, oestrus; EMet, early metoestrus; LMet, late metoestrus;Di, dioestrus.

The results of our analysis show that FAAH activity in the mouse uterus changed as function of the oestrous cycle stages, and that FAAH protein content paralleled the enzyme activity (Fig. 1). In particular, the highest FAAH activity and protein content were recorded at proestrus, followed by a progressive decrease at oestrus and metoestrus; finally, dioestrus was characterized by the lowest values for both parameters, down to ~20% or ~50% of the proestrus values respectively (p < 0.01 for dioestrus *versus *proestrus in both cases). In a variety of tissues high FAAH hydrolyzing activity has been associated with low AEA levels and *viceversa*, both in the central nervous system [14] and in the periphery [15]. In particular, in a previous study we have shown that uterine FAAH activity inversely correlates with uterine AEA levels, so that higher FAAH implicated lower AEA [11]. On this background, we can speculate that the levels of AEA are very likely to be low at proestrus, and that they progressively increase with a maximum at dioestrus.

Serum concentrations of estradiol (E2) and progesterone (P) in the CD-1 mouse oestrous cycle have been carefully analysed in previous studies [16,17]. It has been shown that there are two peaks in both E2 and P endogenous concentrations. The highest E2 level occurs in proestrus, followed by a smaller peak in late metoestrus. The serum concentration of P exhibits a small peak in early oestrous and a large broad peak that spans both metoestrus and dioestrus. Following successful fertilization, both E2 and P are required for uterine receptivity and implantation.

By overlapping the literature data on the endogenous levels of E2 and P with the present analysis of FAAH, it appears that the highest levels of E2 and the lowest levels of P coincide with the highest FAAH activity (i.e., the lowest local levels of AEA); instead, a low FAAH activity (i.e., a high local level of AEA) parallels decreasing and increasing E2 and P concentrations, respectively. Altogether, these data suggest that in the non-pregnant uterus endogenous E2 and P exert opposite effects on FAAH activity, the first hormone being stimulatory and the second inhibitory. This seems at variance with the mouse pregnant uterus, where treatment with exogenous E2 and P down-modulates FAAH activity [4].

Among several effects that variation of FAAH activity and the resulting different local AEA levels could exert on the uterus, the action on the smooth muscle seems of particular interest. It has been demonstrated that endocannabinoids exert a potent and direct relaxant effect on human pregnant myometrium [18] and coordinate oviductal smooth muscle contraction during embryo transport [3]. Moreover, proliferation and apoptosis of uterine tissues are also possible targets of endocannabinoid action [19-22]. In fact, the pro-apoptotic activity of AEA has been documented in several cells and tissues [21], including mouse blastocyst [2]. It is well established that one of basic events of uterine cellular changes is the systemic cell turnover, that consists of cell renewal by proliferation and cell death by apoptosis. While uterine epithelial proliferative indices are highest on the proestrus day, epithelial apoptotic indices are lowest on the same day. A reverse pattern was demonstrated between uterine cell proliferation and apoptosis on the oestrous day [23-25]. The increased uterine cell proliferation has been correlated with increasing ovarian E2 secretion on the proestrus day [26], while increased uterine epithelial apoptosis on the oestrous day has been attributed to decline in serum E2 levels [27]. In addition, a number of studies in humans also showed that preovulatory ovarian estrogen secretion stimulates proliferation of uterine glandular epithelial cells, especially in functional endometrium, during proliferative phase. A rapid increase in the apoptotic index was noted in both epithelium and stroma during the last days of the cycle (late secretory phase), with a maximum index on the second day of menstruation [28]. On the basis of our study, it is possible to speculate that at proestrus low levels of AEA could favour the proliferation of uterine tissues, while at metaestrus-dioestrus high levels of AEA contribute to programmed cell death.

In conclusion, we report here the analysis of FAAH activity and protein content in the mouse uterus, showing variations as a function of the oestrous cycle stages which can be related to changes of sex steroid levels and tissue remodelling. The present data may form the basis for further studies on the action of endocannabinoid signalling in this organ, and on its impact on reproductive physiology.

## Competing interests

The author(s) declare that they have no competing interests.

## Authors' contributions

FGK and NB have produced most of the results and have been involved in drafting of the manuscript. MDF and MM have made equal contributions to conception and design and analysis and interpretation of data as well. All authors have given final approval of the version of the manuscript to be published.
